# *Pig-L* mediates virulence, biofilm formation, and oxidative stress tolerance in *Clostridioides difficile*

**DOI:** 10.3389/fmicb.2025.1691769

**Published:** 2025-10-30

**Authors:** Yumei Cheng, Huilin Hu, Tingyu Huang, Xiang Luo, Fahui Chen, Peng Shi, Weihao Ma, Yingjun Lu, Siyu Lan, Guzhen Cui, Xiaolan Qi, Ya-Jun Liu, Wei Hong

**Affiliations:** ^1^Department of Critical Care Medicine, The Affiliated Hospital of Guizhou Medical University, Guiyang, China; ^2^Key Laboratory of Endemic and Ethnic Diseases, Ministry of Education and School/Hospital of Stomatology, Guizhou Medical University, Guiyang, China; ^3^Clinical Medical College, Guizhou Medical University, Guiyang, China; ^4^School/Hospital of Stomatology, Guizhou Medical University, Guiyang, Guizhou, China; ^5^Key Laboratory of Microbiology and Parasitology of the Education Department of Guizhou, Guizhou Medical University, Guiyang, China; ^6^Collaborative Innovation Center for Prevention and Control of Endemic and Ethnic Regional Diseases Co-Constructed by the Province and Ministry, Guiyang, China; ^7^CAS Key Laboratory of Biofuels, Shandong Provincial Key Laboratory of Synthetic Biology, Shandong Engineering Laboratory of Single Cell Oil, Qingdao Institute of Bioenergy and Bioprocess Technology, Chinese Academy of Sciences, Qingdao, China; ^8^Shandong Energy Institute, Qingdao, China; ^9^Qingdao New Energy Shandong Laboratory, Qingdao, China

**Keywords:** *Clostridioides difficile*, phosphatidylinositol glycosyltransferase L class gene (*pig-L* gene), gene deletion, virulence, biofilm, oxidative stress tolerance

## Abstract

**Background:**

*Clostridioides difficile* infection (CDI) represents a significant global public health concern. The Phosphatidylinositol Glycan Class L (*pig-L*) gene in *C. difficile* encodes an enzyme critical for the biosynthesis of Glycosylphosphatidylinositol (GPI) anchor, which play a vital role in bacterial surface protein localization and function.

**Methods:**

To investigate the role of *pig-L* in *C. difficile* pathogenesis, we utilized CRISPR-Cas9 gene editing to generate a *pig-L* knockout strain and a complementation strain in the wild-type (WT) background. Phenotypic characterization of these strains was performed through a suite of assays, including virulence assays, biofilm formation assays, oxidative stress sensitivity testing, and antimicrobial susceptibility testing. Proteomics analysis was conducted to identify differentially expressed proteins in the knockout strain.

**Results:**

Deletion of the *pig-L* gene resulted in a significant reduction in *C. difficile* virulence, decreased biofilm formation, and increased susceptibility to oxidative stress. Proteomic analysis revealed significant alterations in protein expression, with 170 proteins exhibiting upregulation and 101 proteins demonstrating downregulation in the knockout strain. Complementation of the *pig-L* gene partially restored the phenotypes observed in the deletion strain.

**Conclusion:**

These findings demonstrate that the *pig-L* gene functions as a crucial regulator of *C. difficile* virulence, biofilm formation, and peroxide resistance. Targeting the *pig-L* gene or its downstream effectors represents a promising avenue for the development of novel therapeutic strategies to effectively control *C. difficile* infection.

## 1 Introduction

*Clostridioides difficile* (*C. difficile*) is an intestinal pathogen known for its production of toxins and is the etiological agent of *C. difficile* infection (CDI) (Schäffler and Breitrück, 2018). It possesses flagella for motility and reproduces through sporulation, primarily spreading via the fecal-oral route (Zeng et al., 2022). This bacterium is a primary causative agent of antibiotic-associated diarrhea, accounting for about 50–75% of such cases. Moreover, it plays a significant role in antibiotic-associated colitis and pseudomembranous colitis, responsible for 50–75% and 95–100% of these conditions, respectively (Nakao et al., 2020).

The primary clinical symptoms of CDI include diarrhea, abdominal pain, and fever. Severe cases may lead to pseudomembranous colitis and potentially progress to toxic megacolon, bowel perforation, septic shock, or even death (Abbas and Zackular, 2020). CDI is associated with prolonged hospital stays, high recurrence and mortality rates, posing a severe risk to hospitalized patients. The emergence of hypervirulent strains such as ribotype 027/BI/NAP1 has intensified these challenges. According to data from the U.S. Centers for Disease Control and Prevention, there are approximately 453,000 CDI cases annually in the United States, resulting in nearly 29,000 deaths and healthcare expenditures reaching $4.8 billion [Centers for Disease Control and Prevention (U.S.), 2019]. The European Union faces an estimated annual economic burden of up to €300 million due to CDI (Wiegand et al., 2012). In China, research on *C. difficile* has significantly increased over the past decade. Meta-analyses reveal that up to 14% of patients with diarrhea are found to carry toxigenic *C. difficile* (Wen et al., 2023). Additionally, cross-regional studies indicate that 10% of patients with diarrhea are affected by CDI (Jin et al., 2017). These findings underscore that CDI is a critical pathogen that significantly impacts human health and imposes substantial burdens on healthcare systems.

The Glycosylphosphatidylinositol (GPI) anchor is a complex glycolipid compound that covalently anchors proteins to the outer surface of the cell membrane, playing roles in various biological and pathological processes (Guo and Kundu, 2024). The common core structure of GPI includes phosphatidylinositol, GlcN, three mannose molecules, and an ethanolamine phosphate. The biosynthesis of GPI occurs on the membrane of the endoplasmic reticulum (ER) (Watanabe et al., 1999). In eukaryotic cells, proteins on the cell surface are connected to the cell membrane via GPI anchors. In yeast, the biosynthesis of the GPI anchor involves 11 steps (Müller, 2011; Komath et al., 2018). The gene phosphatidylinositol glycan class L (*pig*-L) encodes the N-deacetylase enzyme Gpi12p, which catalyzes the second step in GPI anchor biosynthesis: the deacetylation of N-acetylglucosaminyl-phosphatidylinositol (GlcNAc-PI) to form glucosaminyl (GlcN)-PI (Watanabe et al., 1999). Mutations in the *pig-L* gene in eukaryotes can lead to defects in the GPI anchor biosynthetic pathway, affecting the precise localization and function of cell surface proteins, thereby altering cellular functions. In CHO-K1 cells, *pig-L* encodes a membrane protein with 252 amino acids, predominantly located on the cytoplasmic side (Nakamura et al., 1997). Abnormalities in *pig-L* gene function are associated with various genetic disorders, such as CHIME syndrome, Fryns syndrome, and syndromes related to intellectual disability and epilepsy. These diseases present symptoms including delayed psychomotor development, seizures, and possibly elevated alkaline phosphatase levels (Ceroni et al., 2018).

In prokaryotes, studies on homologous genes of *pig-L* are relatively limited. In *Pseudomonas aeruginosa*, the *dnpA* gene from the *pig-L* family deacetylase is associated with fluoroquinolone tolerance (Liebens et al., 2014). The receptor for *Clostridium septicum* alpha toxin, a GPI-anchored protein, is also involved (Gordon et al., 1999). In *Clostridioides difficile*, a gene sequence homologous to *pig-L* is present (CD23150), but its function in this organism and its impact on virulence remain unclear. This study plans to use CRISPR-Cas9 gene editing technology to obtain Δ*pig-L* mutant strains and their complemented strain (::*pig-L*), comparing the phenotypes of wild-type and mutant strains to elucidate the function of the *pig-L* gene in *Clostridioides difficile*.

## 2 Materials and methods

### 2.1 Strains and culture conditions

The *Clostridioides difficile* CD630 strain was acquired from the American Type Culture Collection (ATCC BAA-1382). The ∆*pig-L* mutant and the complemented ::*pig-L* strains of *C. difficile* were constructed in this study. For culturing, *C. difficile* strains were grown in Brain Heart Infusion (BHI) agar medium supplemented with yeast extract in a COY anaerobic workstation (Model: COY-7200220). *Escherichia coli* CA434 and NEBExpress (New England BioLabs, Beijing) were cultured using Luria-Bertani (LB) medium. Vero cells were maintained in DMEM complete culture medium under 5% CO_2_ conditions in a CO_2_ incubator. To prepare the BHI agar medium, brain heart infusion powder (38.5 g/L), L-cysteine (1 g/L), and yeast extract (5 g/L) were dissolved in 1 liter of double-distilled water, with agarose at 1.5% added as needed to obtain a solid medium (Zhou et al., 2022). For LB medium preparation, tryptone (10 g/L), sodium chloride (NaCl, 10 g/L), and yeast extract (5 g/L) were dissolved in 1 liter of double-distilled water. To prepare the solid medium, agarose at 1.5% was added to this solution. Reagents such as L-cysteine, yeast extract, tryptone, sodium chloride, tetracycline, amoxicillin, ampicillin, vancomycin, erythromycin, metronidazole, clindamycin, and norfloxacin were sourced from Solarbio Biotechnology Co., Ltd. (Beijing). Restriction enzymes Btg ZI (R0703S), Not I-HF (R3189S), and Bam HI-HF (R3136V) were obtained from New England BioLabs (Beijing). DNA recovery kits, plasmid extraction kits, and ClonExpress Multis One Step Cloning reagents were purchased from Vazyme Biotech Co., Ltd. (Nanjing). Gene sequencing services were provided by Sangon Biotechnology Co., Ltd. (Shanghai).

### 2.2 Sequence homology analysis

The protein multiple sequence alignment process was conducted as follows: Initially, the target gene (e.g., *Clostridium difficile* AM180355.1, CD630_23150) was searched in NCBI^1^ to obtain its amino acid sequence. Subsequently, Protein BLAST^2^ was used with the refseq_select database to identify homologous sequences, filtering for sequences originating from typical strains and downloading all FASTA files. The integrated sequences were submitted to CLUSTALW^3^ (Thompson et al., 2003) for multiple sequence alignment, generating an *.aln file. This was followed by using Jalview 2.11.4.0 software (Waterhouse et al., 2009) to adjust the layout and output SVG vector-editable sequence comparison results. Further analysis utilized TMHMM^4^ for predicting transmembrane domains of the target protein. Finally, Affinity Designer 2.4.0 was employed to adjust image quality, producing a visualization PDF map that includes transmembrane structure domains (TM), conserved residues (marked by asterisks/black shadow), and similarity (indicated by asterisks/colons/grey shadows).

### 2.3 Plasmid construction

The targeting plasmid for the *pig-L* gene contains a segment driven by the small RNA promoter (PsRNA) that recognizes *pig-L* crRNA, flanked by homologous arms upstream and downstream of the *pig-L* gene (Hong et al., 2018). The construction steps are as follows: (1) Using pJZ23 as the backbone vector, the PsRNA promoter+crRNA fragment amplified from pSH12 was ligated to Btg ZI linearized pJZ23 to obtain plasmid pHTY4. (2) Employing the *C. difficile* CD630 genome as a template, PCR amplification of the upstream and downstream homologous arms of the *pig-L* gene was performed, followed by their ligation into Not I linearized pHTY4 backbone, resulting in plasmid pHTY5 (Zhang et al., 2020). The pHTY5 plasmid, facilitating *pig-L* gene knockout, was purified using a plasmid extraction kit. To construct the *pig-L* complementation strain, primers HW2125/HW2156 were used to amplify the *pig-L* promoter+gene fragment. This amplified segment was then recombined with BamH I linearized pMTL82151 vector to generate the *pig-L* gene complementation plasmid pHTY6 (Yang et al., 2025).

### 2.4 Bacterial growth rate determination

Bacterial strains WT, Δ*pig-L*, and :: *pig-L* were individually streaked for single colonies on solid agar plates. Single colonies were then inoculated into Brain Heart Infusion Supplemented (BHIS) liquid medium and anaerobically cultured at 37 °C until reaching the logarithmic growth phase (OD600 ≈ 0.6). Cultures were subsequently transferred to fresh BHIS medium with a 1% inoculum, with three biological replicates per strain. Growth was monitored by measuring OD600 at 3-h intervals using a spectrophotometer (Varioskan LUX, Thermo Fisher Scientific, United States). Growth curves were generated by plotting OD600 values against incubation time (h), and growth rate characteristics were analyzed.

### 2.5 Antibiotic susceptibility testing

Antibiotic susceptibility of strains WT, Δ*pig-L*, and :: *pig-L* to metronidazole, vancomycin, amoxicillin, clindamycin, cefoxitin, norfloxacin, ampicillin, and erythromycin was determined using the plate dilution method according to the guidelines of Clinical and Laboratory Standards Institute (CLSI) (Lewis et al., 2025). Antibiotics were solubilized in a proper solution and sterile filtered (e.g., tetracycline, norfloxacin, and cefoxitin were solubilized in hydrochloric acid, acetic acid, and dimethyl sulfoxide, respectively. Other antibiotics were dissolved in sterile water) BHIS medium, melted and cooled to 45 ~ 50 °C, was mixed with the stock antibiotic solutions to achieve final concentrations of 128 μg/mL, 64 μg/mL, 32 μg/mL, 16 μg/mL, 8 μg/mL, 4 μg/mL, 2 μg/mL, 1 μg/mL, 0.5 μg/mL, and 0.25 μg/mL. A negative control, consisting of BHIS medium without antibiotic addition, was also included. Colonies of each mutant strain were inoculated into 5 mL of BHIS liquid medium and incubated at 37 °C under anaerobic conditions until the optical density at 600 nm (OD600) reached 0.5. Following this, 2 μL of the bacterial culture was spotted onto the surface of the agar plates, with three replicates per strain. The plates were incubated for 18–24 h, and the resulting growth was assessed (Zhao et al., 2020).

### 2.6 Vero cell cytotoxicity assay

Strains WT, Δ*pig-L*, and :: *pig-L* were cultured in Brain Heart Infusion Supplemented (BHIS) medium at 37 °C under anaerobic conditions until OD600 reached 1.0. African green monkey kidney cells (Vero cells) served as the experimental model, grown in Dulbecco’s Modified Eagle Medium (89% DMEM) supplemented with 10% fetal bovine serum and 1% antibiotics (10 mg/mL streptomycin and 10,000 U/mL penicillin). Cells were cultured to approximately 60% confluence. Vero cells were then washed three times with phosphate-buffered saline (PBS), followed by a 24-h incubation in DMEM at 37 °C. For each strain, 1 mL of culture was centrifuged at 8,000 rpm for 6 min. The supernatants were filter-sterilized using a 0.22 μm membrane and serially diluted in BHIS medium from 5 × 10^1^ to 5 × 10^8^-fold. Both diluted and undiluted supernatants (200 μL each) were added to Vero cells, which were then incubated overnight at 37 °C. Cell morphology was examined using an optical microscope to identify the highest dilution causing cytopathic effects in Vero cells (Yang et al., 2025).

### 2.7 Biofilm formation assay

Overnight cultures were diluted to a 1:200 concentration in fresh BHIS medium. Following thorough mixing, 2 mL of the diluted culture was dispensed into each well of a 24-well plate, with four replicates per sample and a blank control consisting of BHIS medium alone. The plate was then incubated at 37 °C under anaerobic conditions for 48 h after sealing. Upon incubation completion, the surface growth was carefully removed from each well using a micropipette, avoiding contact with the well walls. Subsequently, 1 mL of 0.2% crystal violet solution was added to each well for fixation and staining, followed by a 1-h incubation at 37 °C. After removal of the stain, the wells were washed three times with PBS until the wash solution was nearly colorless. The plate was then air-dried at room temperature and photographed. Finally, 1 mL of anhydrous ethanol was added to each well and incubated at room temperature for 15 min to fully dissolve the bound crystal violet.

## 3 Results

### 3.1 Multiple sequence alignment

**Figure 1:**
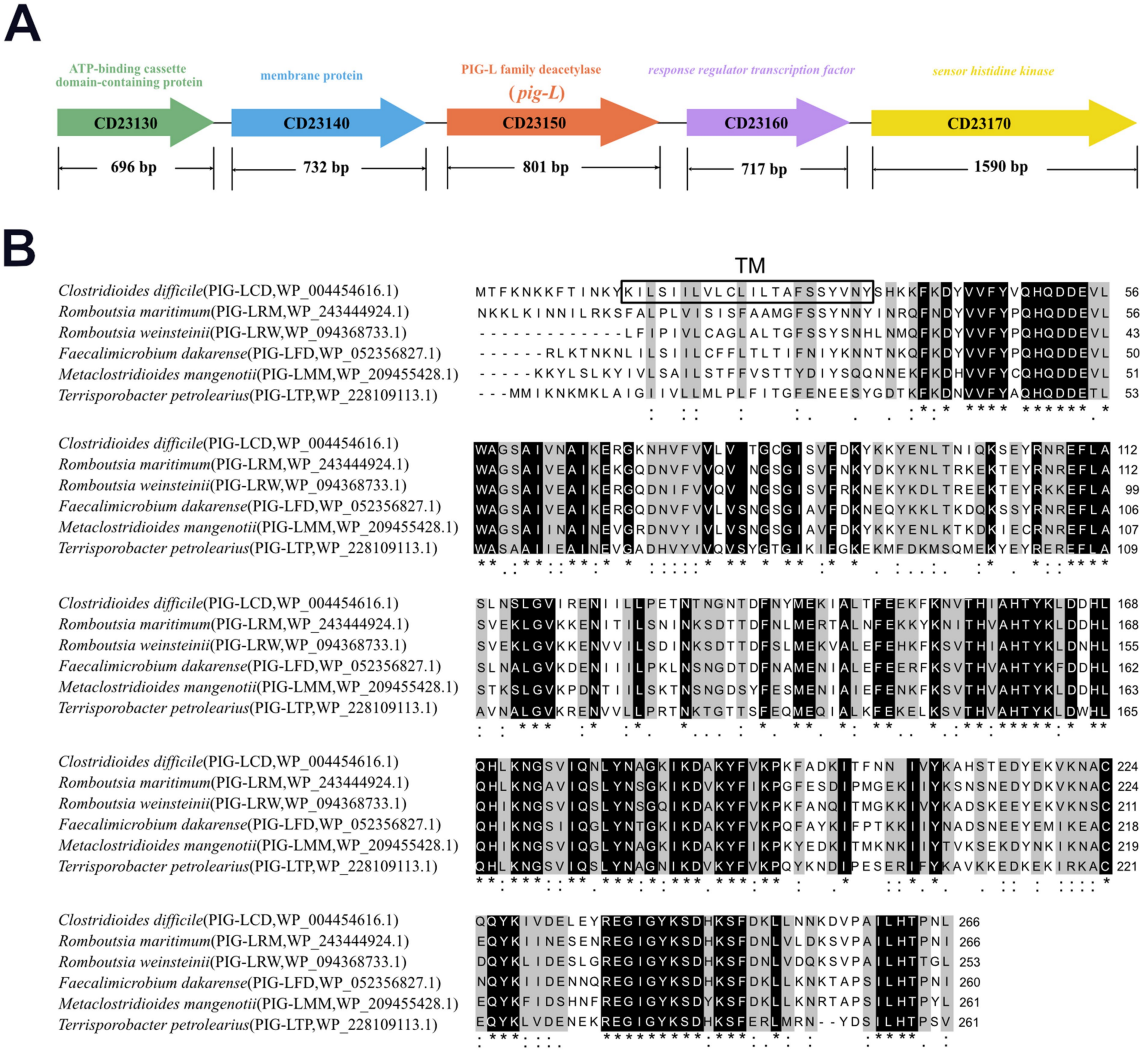
Multiple sequence alignment of *Clostridioides difficile* PIG-L with PIG-L family proteins from other bacteria. **(A)** Genomic context of the pig-L gene. **(B)** The PIG-L protein from *Clostridioides difficile* was aligned with homologous sequences from *Romboutsia maritima, Romboutsia weinsteinii, Faecalimicrobium dakarense, Metaclostridioides mangenoti,* and *Terrisporobacter petrolearius* using Clustal Omega. The boxed region indicates the predicted transmembrane (TM) domain from *Clostridioides difficile* PIG-L. Black-shaded areas and asterisks denote identical amino acids, while colons, single dots, and gray-shaded areas represent similar amino acids. An asterisk (*) signifies fully conserved sites, a colon (:) indicates strongly similar residues, and a single dot (.) denotes weakly similar residues.

The *pig-L* gene (CD23150) is 801 base pairs (bp) in length and encodes a 267 amino acid (aa) protein. It is flanked by the genes CD23140 and CD23160, which encode a membrane protein and a response regulator transcription factor, respectively (Figure 1A). To investigate the potential structural and functional features of the PIG-L (WP_004454616.1) protein from *Clostridioides difficile*, its amino acid sequence was aligned with homologous proteins from related bacterial species using Clustal Omega (Sievers et al., 2011), *Romboutsia weinsteinii* (PIG-LRW_WP_094368733.1), *Faecalimonas dakanense* (PIG-LFD_WP_052356827.1), *Metaclosteridium mangenotii* (PIG-LMM_WP_209455428.1), and *Terrisporobacter petrolearius* (PIG-LTP_WP_228109113.1). The multiple sequence alignment (Figure 1B) revealed a high degree of amino acid conservation among these homologous proteins, particularly in several distinct regions indicated by shaded residues and asterisks. This high conservation suggests that these regions are functionally and structurally important across these species. A predicted transmembrane (TM) region was identified near the N-terminus of the protein (amino acids ~22–35, highlighted with a grey background in Figure 1B), indicating its likely insertion into a cellular membrane. Despite overall conservation, some variations in amino acid sequences were observed between the different bacterial species, which may account for species-specific functional adaptations. The consistent presence of the TM domain across these clostridial and related species implies a conserved membrane-associated role for this protein family.

### 3.2 Plasmids construction

**Figure 2:**
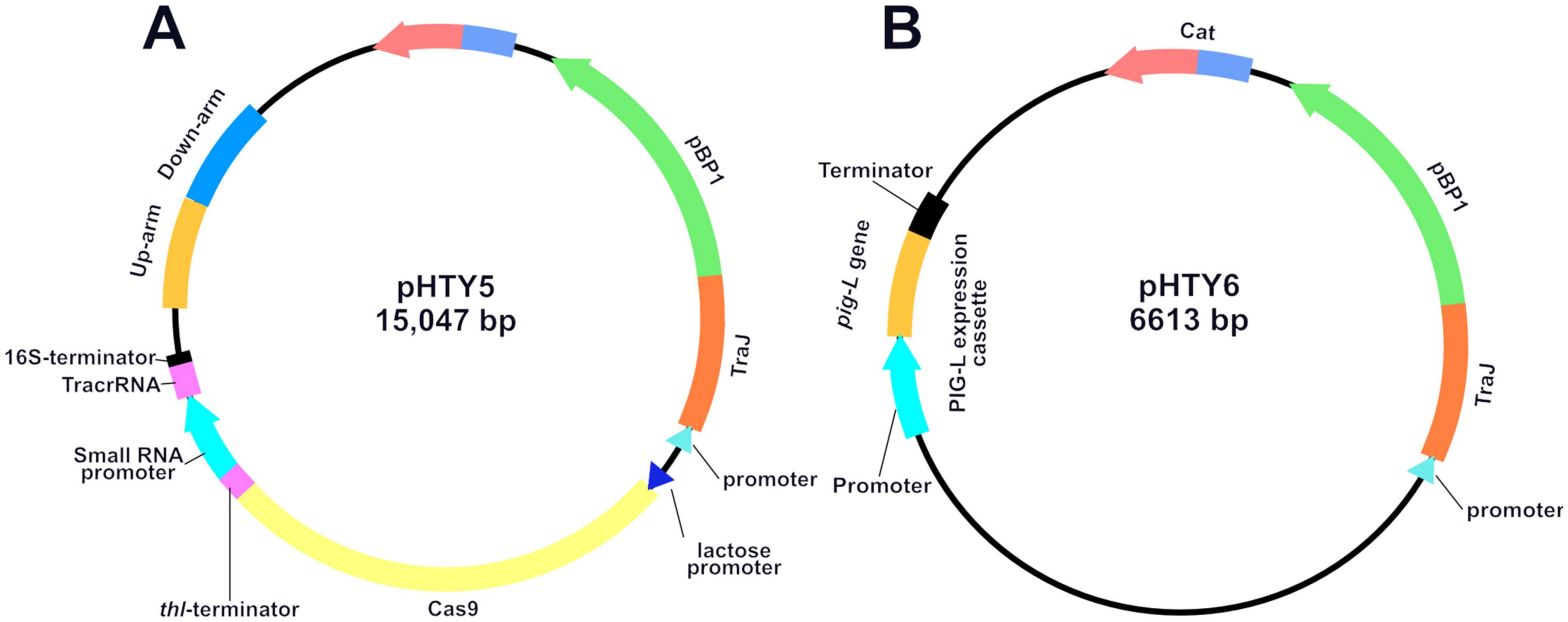
Plasmid Maps of pHTY5 **(A)** and pHTY6 **(B)**. Schematic diagrams of the *Clostridioides difficile pig-L* gene knockout plasmid pHTY5 and complementation plasmid pHTY6.

The [PsRNA promoter + crRNA] sequence was amplified from pSH12, yielding a 381 bp target fragment. This fragment was then ligated into the pJZ23 vector (Zhang et al., 2020) at the Btg ZI single restriction site. Gel electrophoresis (821 bp) confirmed successful recombination and ligation, showing successful construction of pHTY4. Subsequently, using the wild-type (WT) genome as a template, PCR amplification yielded a 645 bp upstream homologous arm and a 722 bp downstream homologous arm. This homologous arm fragment was ligated into the Not I-HF linearized pHTY4 to generate *pig-L* targeting plasmid pHTY5 (Figure 2A). Next, BamH I linearized the pMTL82151 vector, and the *pig-L* gene, along with its native promoter, were combined by using the TEDA method (Xia et al., 2019), yielding the *pig-L* complementation vector, pHTY6 (Figure 2B).

### 3.3 Screening for *pig-L* gene mutant and plasmid curing

**Figure 3:**
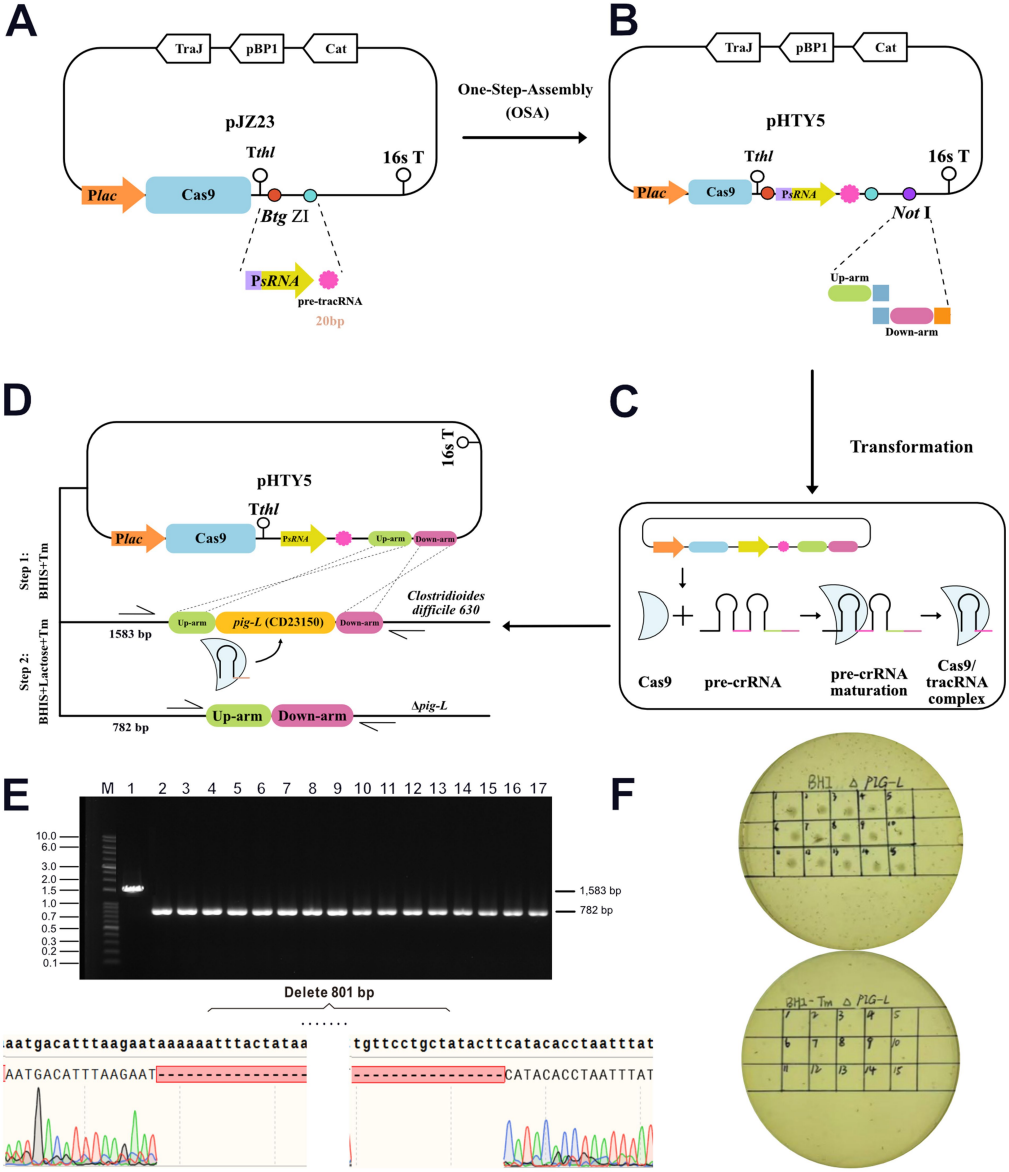
Generation and validation of the ∆*pig-L* mutant strain. **(A)** The pre-tracRNA expression cassette was inserted into the pJZ23 plasmid backbone. **(B)** The pHTY5 targeting plasmid was constructed by incorporating the upstream and downstream homology arms of the *pig-L* gene into the vector from the previous step. **(C)** Expression and assembly of the pHTY5 plasmid into the targeting effector complex. **(D)** A two-step selection process to isolate the Δ*pig-L* mutant in *C. difficile*. Step 1, *C. difficile* transformants harboring a pHTY5 targeting plasmid were cultured in BHIS medium supplemented with thiamphenicol (Tm) (BHIS+Tm). The presence of Tm maintained the pHTY5 plasmid within the transformants. Step 2: The transformants were plated onto solid BHIS+Lactose+Tm agar. Lactose induced Cas9 protein expression, leading to the formation of a mature Cas9/tracrRNA ribonucleoprotein complex that specifically targeted the *pig-L* gene. Homologous recombination between the cleaved genomic DNA and the pHTY5 homology arms resulted in the generation of Δ*pig-L* knockout mutants. Transformants that failed to undergo homologous recombination succumbed to a genomic DNA double break. **(E)** Verification of the ∆*pig-L* mutant was conducted via colony PCR. All examined colonies (*n* = 16, upper panel) exhibited the ∆*pig-L* gene deletion, evidenced by the amplicon size difference (1814 bp for wild-type, 875 bp for ∆*pig-L*). Subsequent gene sequencing confirmed the deletion of the *pig-L* gene within the ∆*pig-L* mutant (lower panel). **(F)** Curing of the targeting plasmid from the ∆*pig-L* mutant was assessed. Thiamphenicol resistance was tested for the first generation (upper panel) and again after 10 consecutive passages (lower panel).

Wild-type (WT) *C. difficile* was utilized as the mother strain. Transformants harboring the recombinant pHTY5 plasmid were selected on Lac-BHIS medium supplemented with thiamphenicol (Tm) (Figures 3A–D). PCR identification of 16 randomly selected single colonies was performed using primers HW2066/HW2067 (Supplementary Table S1). All tested strains exhibited a reduction in PCR product size consistent with the designed knockout sequence (801 bp). Further gene sequencing confirmed the successful deletion of the *pig-L* gene sequence from the *Clostridioides difficile* chromosome, resulting in a gene knockout efficiency of 100% (Figure 3E). Following selection of the mutant strain in Lac-BHI-Tm medium, the pHTY5 plasmid was cured from the deletion mutant by continuous subculturing in BHIS medium, yielding a stable ∆*pig-L* mutant strain (Figure 3F).

### 3.4 Analysis of *pig-L* gene expression and growth kinetics

**Figure 4:**
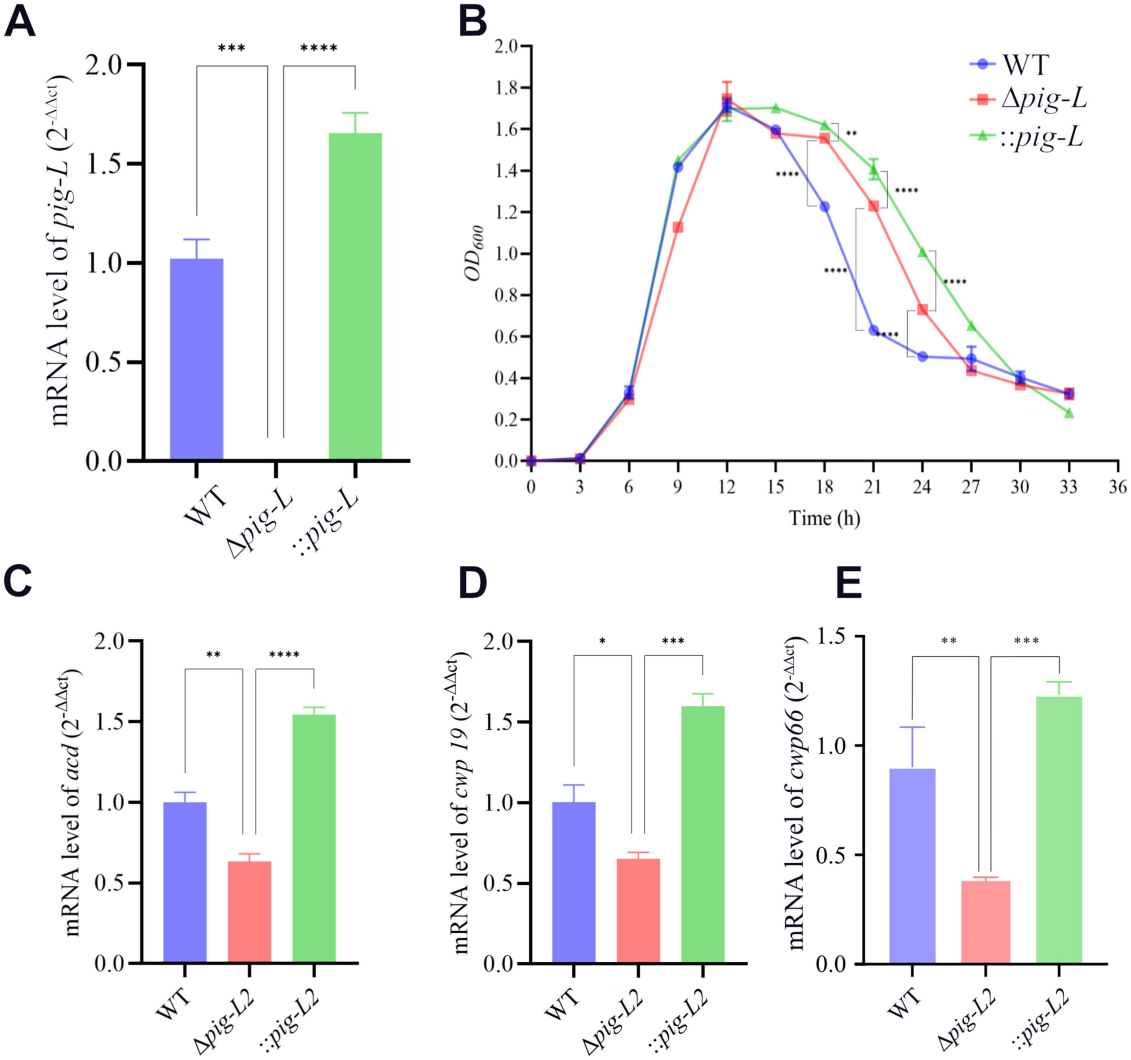
Expression of the *pig-L* gene and its effect on bacterial growth in WT, ∆*pig-L*, and :: *pig-L* strains. **(A)** The expression level of the *pig-L* gene in WT, ∆*pig-L*, and :: *pig-L* strains. **(B)** Determination of the growth curves. The horizontal coordinate represents time in hours, and the vertical coordinate represents the optical density at OD600 nm. **(C–E)** The expression levels of the *acd*, *cwp19*, and *cwp66* genes are in WT, Δ*pig-L*, and :: *pig-L* strains. Statistical significance is denoted as follows: *: *p* <0.05: **: *p* < 0.01; ***: *p* < 0.001; ****: *p* < 0.0001.

To confirm the deletion of the *pig-L* gene at the transcriptional level and to assess its impact on the growth and autolysis of *C. difficile*, we first quantified *pig-L* transcript levels in the WT, ∆*pig-L*, and :: *pig-L* strains. The results demonstrated that *pig-L* expression was undetectable in the ∆*pig-L* mutant, whereas the complemented strain (::*pig-L*) exhibited high levels of expression; both were significantly different compared to the WT strain (Figure 4A). We then characterized the growth curves for the three strains. No significant differences in growth rate were observed among the WT, ∆*pig-L*, and :: *pig-L* strains during the initial growth phases (0–15 h). However, in the decline phase (15–33 h), the rate of autolysis in the ∆*pig-L* strain was markedly reduced compared to the WT strain (*p* < 0.0001). This attenuated autolysis phenotype was also observed in the :: *pig-L* strain. Specifically, at the 21-h time point, the OD600 values for WT, ∆*pig-L*, and :: *pig-L* were 0.6, 1.2, and 1.4, respectively. These values correspond to a cellular autolysis of 65% in the WT, but only 29% in ∆*pig-L* and 18% in :: *pig-L* (Figure 4B). To elucidate the attenuated autolysis observed in the Δ*pig-L* mutant, we quantified the expression levels of the known autolysin genes *acd* (Figure 4C), *cwp19* (Figure 4D), and *cwp66* (Figure 4E) across different mutant strains. Our results demonstrate a significant reduction in the expression of these genes in the Δ*pig-L* mutant (Dhalluin et al., 2005; Wydau-Dematteis et al., 2018; Zhou et al., 2022).

### 3.5 Toxin gene expression and cytotoxicity assay

**Figure 5:**
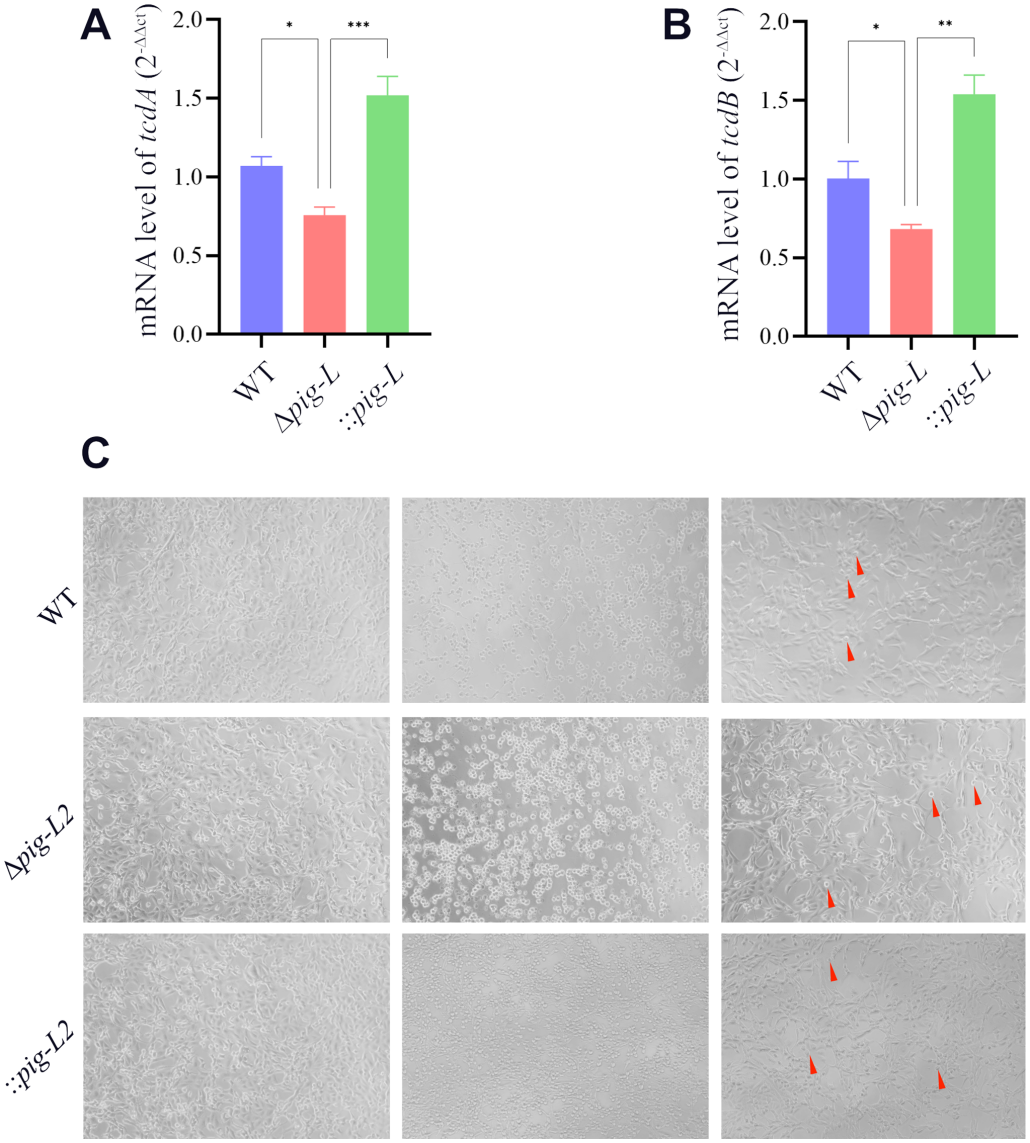
Characterization of WT, ∆*pig-L*, and :: *pig-L* strains. **(A,B)** Quantitative RT-qPCR analysis of *tcdA* and *tcdB* gene expression in the WT, ∆*pig-L*, and :: *pig-L* strains. **(C)** Cytotoxicity assays of the WT, ∆*pig-L*, and :: *pig-L* strains. Vero cells were exposed to PBS (left), undiluted culture supernatants (middle), or diluted supernatants (right) from each strain: WT at 10^−7^ dilution, ∆*pig-L* at 10^−5^ dilution, and :: *pig-L* at 10^−6^ dilution. Red arrows indicate rounded cells. Statistical significance is denoted as follows: *: *p* < 0.05; **: *p* < 0.01; ***: *p* < 0.001.

To investigate the impact of the *pig-L* gene on the cytotoxicity of *C. difficile*, we quantified the expression levels of *tcdA* and *tcdB* toxin genes in the WT, ∆*pig-L*, and:: *pig-L* strains using RT-qPCR. Results demonstrated a significant decrease in *tcdA* (Figure 5A) and *tcdB* (Figure 5B) expression in the ∆*pig-L* mutant compared to the WT strain. Conversely, the :: *pig-L* complemented strain exhibited significantly increased *tcdA* and *tcdB* expression levels relative to the WT strain. Subsequent cytotoxicity assays revealed the highest supernatant dilutions that induced morphological changes (corneoid to round) in Vero cells were 0.5 × 10^-7^ (Figure 5C-WT-Right), 0.5 × 10^-5^ (Figure 5C-∆*pig-L*-Right), and 0.5 × 10^-6^ (Figure 5C-::*pig-L*-Right) for WT, Δ*pig-L*, and :: *pig-L*, respectively. Consequently, the Δ*pig-L* strain exhibited a 100-fold decrease in cytotoxicity compared to the WT, while the :: *pig-L* strain displayed cell cytotoxicity intermediate between WT and Δ*pig-L*, with enhanced cytotoxicity compared to Δ*pig-L*. These findings suggest that deletion of the *pig-L* gene substantially reduces the cytotoxicity of *C. difficile*.

### 3.6 Biofilm formation

**Figure 6:**
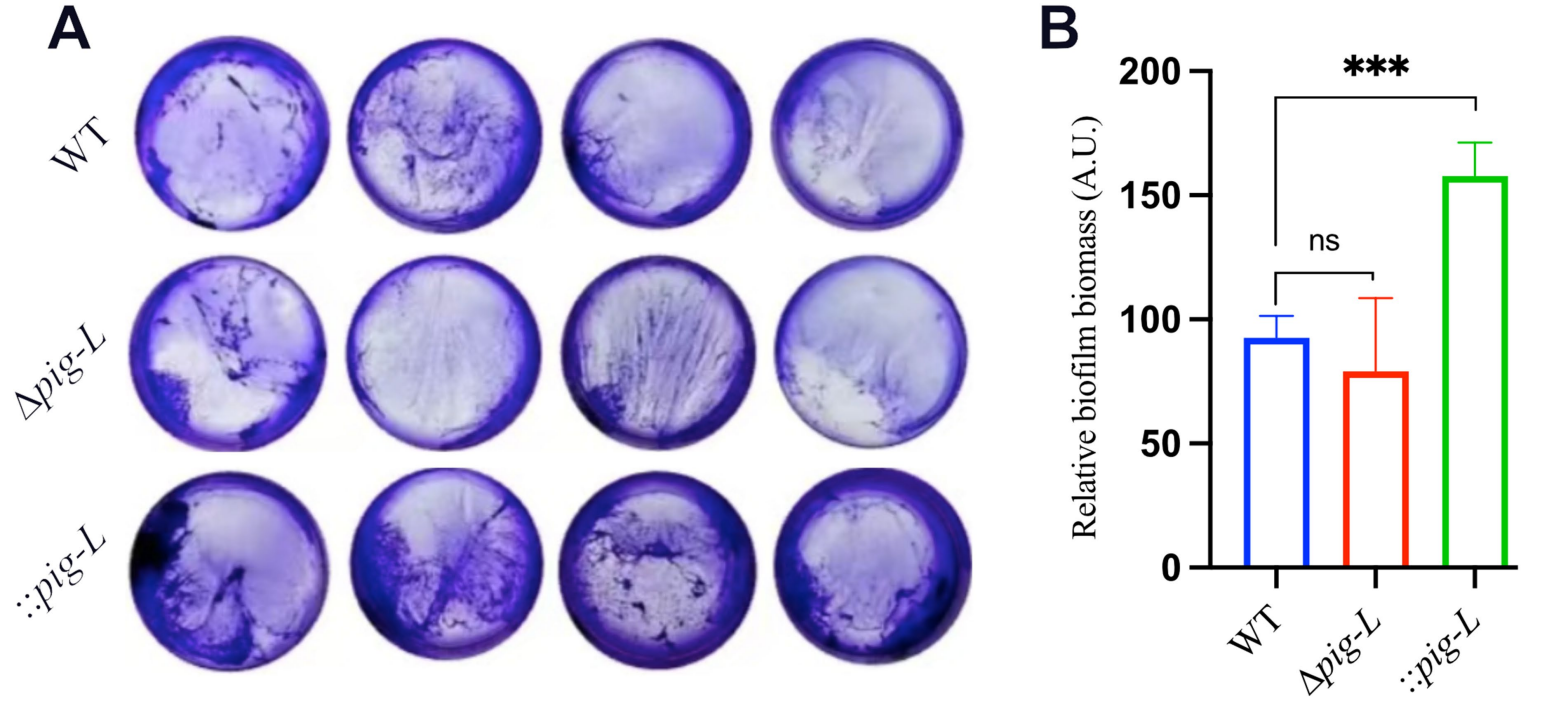
Biofilm formation assays of WT, ∆*pig-L*, and:: *pig-L* strains. **(A)** Biofilm formation of the WT, ∆*pig-L*, and :: *pig-L* strains. **(B)** Quantitative analysis of biofilm formation by the WT, ∆*pig-L*, and :: *pig-L* strains. Statistical significance is denoted as follows: ***: *p* < 0.001; ns: *p* > 0.05.

The *pig-L* gene encodes an N-deacetylase, a key enzyme in the synthesis of Glycosylphosphatidylinositol (GPI) anchors. GPI anchors are complex glycolipids that covalently attach proteins to the outer cell membrane. Cell surface proteins play a critical role in biofilm formation in pathogenic microorganisms (Brauer et al., 2021). Therefore, we investigated the effect of the *pig-L* gene on biofilm formation in *C. difficile*. Results showed that the ∆*pig-L* mutant exhibited reduced biofilm formation compared to the WT strain (Figure 6A). Overexpression of the *pig-L* gene resulted in significantly enhanced biofilm formation in *C. difficile* compared to the WT strain (Figure 6B).

### 3.7 Peroxide tolerance assay

**Figure 7:**
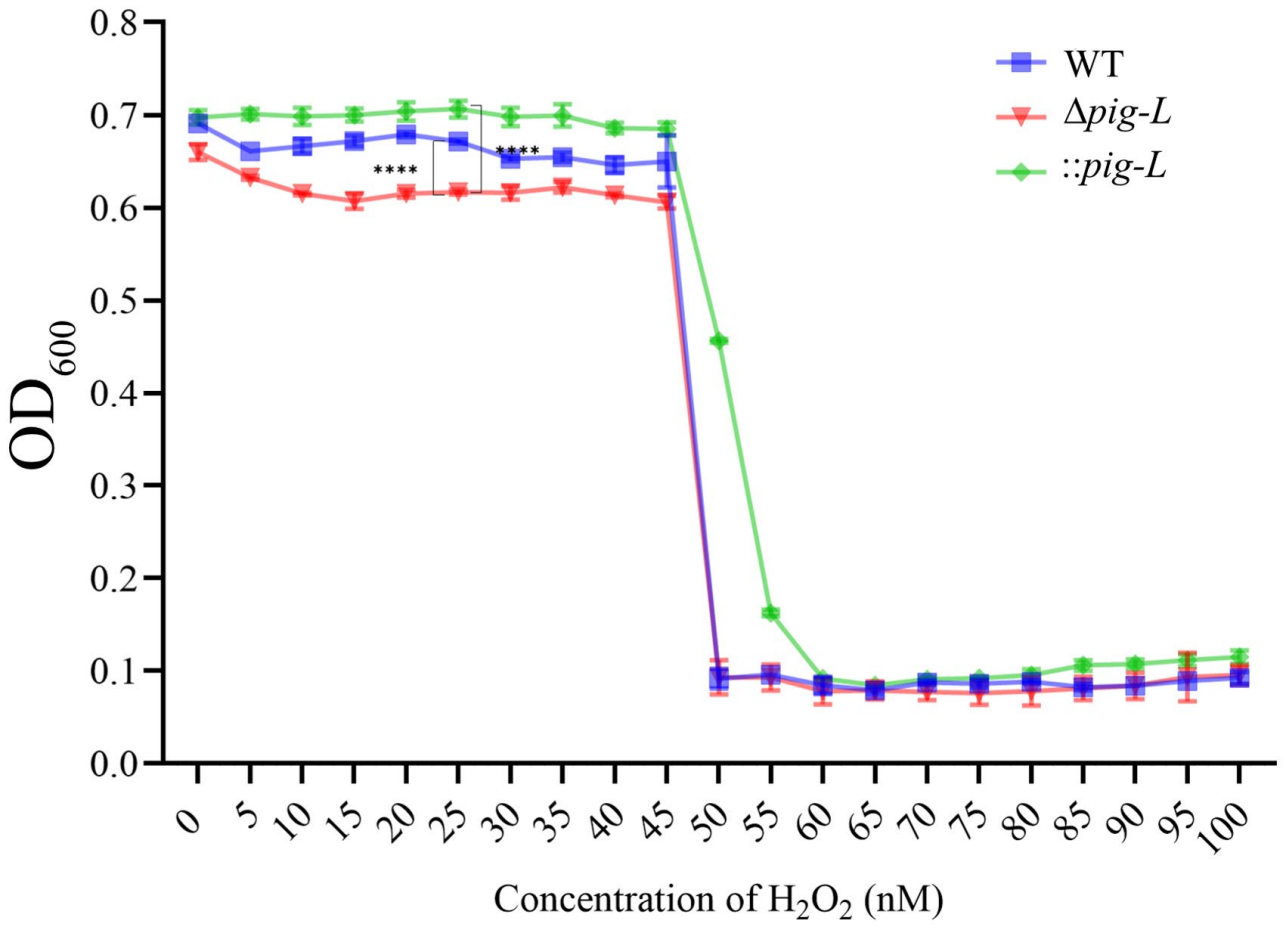
Peroxide tolerance of WT, ∆*pig-L*, and :: *pig-L* Strains. Changes in optical density at 600 nm (OD600) for WT, ∆*pig-L*, and :: *pig-L* strains under various hydrogen peroxide concentrations. Statistical significance is denoted as follows: ****: *p* < 0.0001.

The role of the *pig-L* gene in conferring oxidative stress tolerance to pathogens remains unclear (Watanabe et al., 1999; Pascual and Planas, 2021). To deepen our understanding of the *pig-L* gene’s impact on *C. difficile*’s resistance to reactive oxygen species (ROS), we assessed its influence on peroxide tolerance. Results indicated that compared to WT strains, the ∆*pig-L* mutant showed significantly reduced hydrogen peroxide tolerance; the :: *pig-L* strain exhibited markedly higher peroxide tolerance than WT, particularly at a concentration of 25 nM. Additionally, at higher peroxide concentrations (50 to 55 nM), the :: *pig-L* complemented strain also demonstrated increased hydrogen peroxide tolerance (Figure 7).

### 3.8 Antibiotic susceptibility analysis

**Figure 8:**
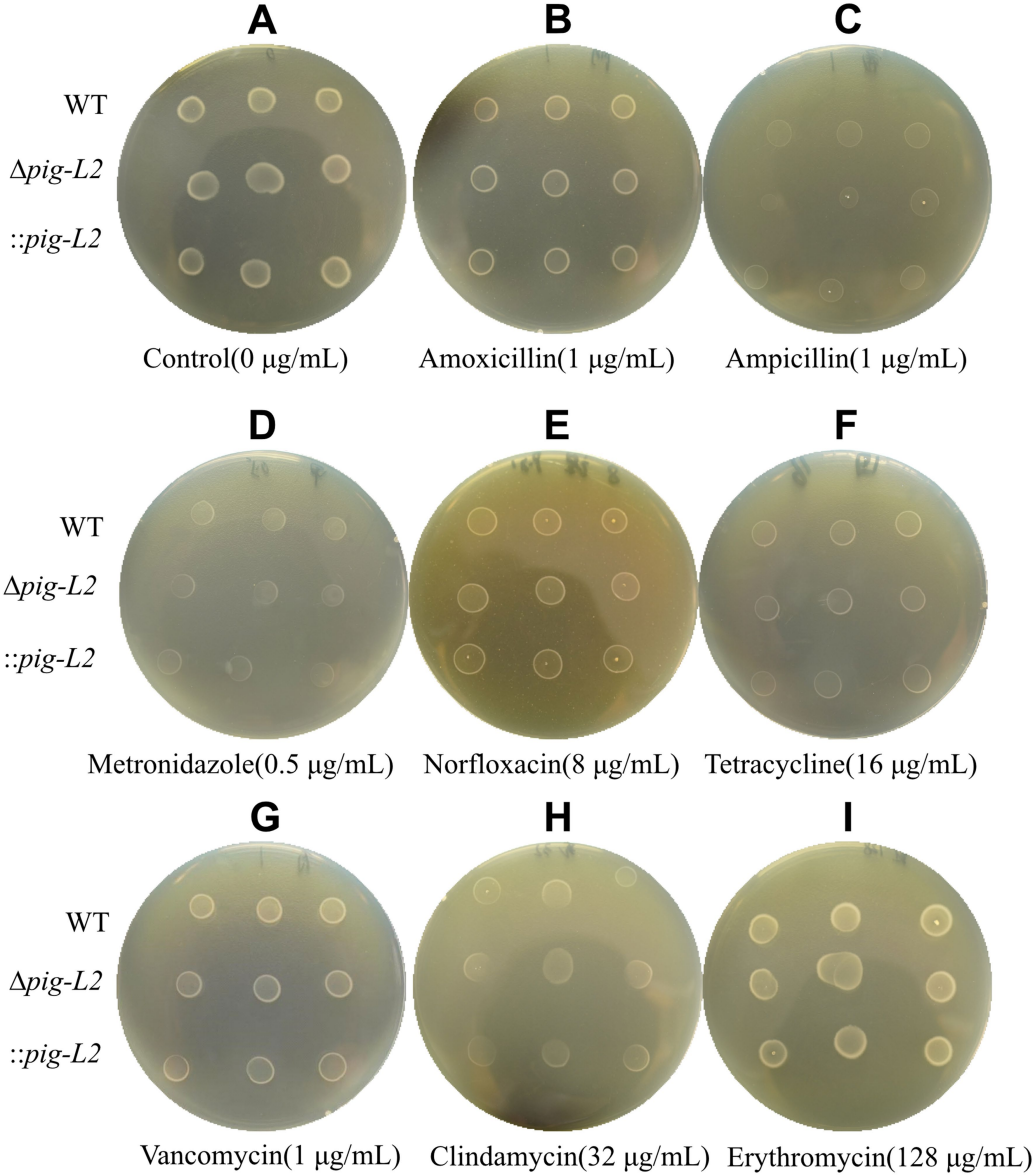
Antibiotic susceptibility test for WT, ∆*pig-L*, and :: *pig-L* Strains. The antibiotics tested include Control **(A)**, Amoxicillin **(B)**, Ampicillin **(C)**, Metronidazole **(D)**, Norfloxacin **(E)**, Tetracycline **(F)**, Vancomycin **(G)**, Clindamycin **(H)**, and Erythromycin **(I)**.

The susceptibility of WT, ∆*pig-L*, and :: *pig-L* strains to various commonly used antibiotics is depicted in Figures 8A–I. Compared with the WT strain, the ∆*pig-L* and:: *pig-L* mutant exhibited no statistically significant alterations in antibiotic resistance across the range of assays performed (Figures 8A–I; Supplementary Table S3).

### 3.9 Protein expression differences

**Figure 9:**
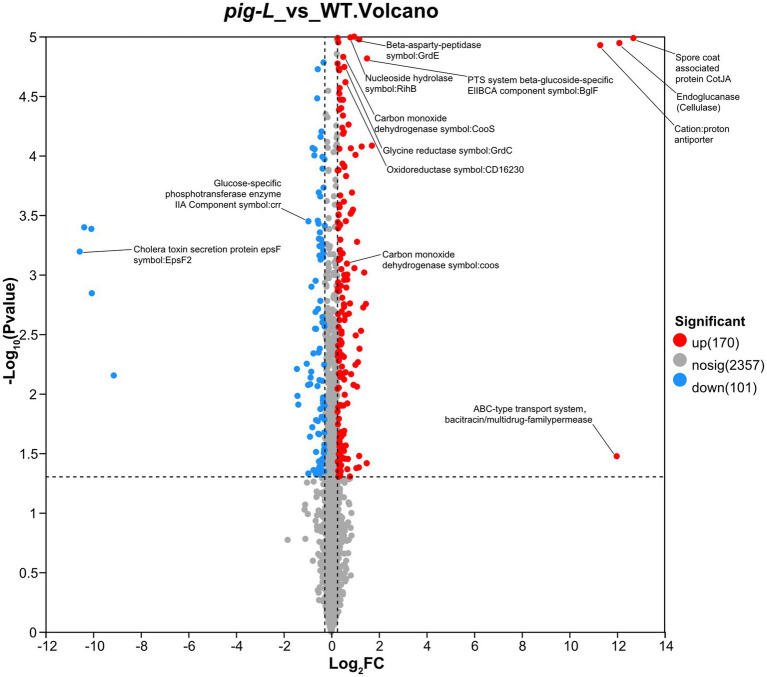
Volcano plot of differentially expressed genes between ∆*pig-L* and WT. Each point represents a gene. The horizontal axis displays the log₂ fold change values (∆*pig-L*/WT), while the vertical axis shows the -log₁₀ of adjusted *p*-values. Red points indicate 170 significantly upregulated genes; blue points represent 101 significantly downregulated genes; gray points denote 2,357 genes with no significant difference in expression between ∆*pig-L* and WT strains.

Proteomic analysis was used to compare gene expression between the ∆*pig-L* mutant strain and the wild type (WT). The volcano plot shows that the ∆*pig-L* group had 170 proteins significantly upregulated, 101 proteins significantly downregulated, and 2,357 proteins with unchanged expression (adjusted *p*-value <0.05, |log₂FC| > 1) (Figure 9). The top 10 notably upregulated proteins include Spore coat-associated protein CotJA, Endoglucanase (Cellulase), ABC-type transport system/bacitracin/multidrug-family permease, Cation:proton antiporter, Putative membrane protein, PTS system beta-glucoside-specific EIIBCA component, FeoA domain, Ferrous iron transport protein B, ABC transporter ribose-specific ATP-binding protein, and D-alanine—D-alanine ligase. The top 10 significantly downregulated proteins include Cholera toxin secretion protein EpsF, DUF3795 domain-containing protein, Phosphatidylglycerol lysyltransferase, Uncharacterized protein, ABC transporter ATP-binding protein, DegV family protein, Spo0E-like sporulation regulatory protein, Ethanolamine transport protein, ABC-type transport system permease, and Uncharacterized protein OS=*Clostridioides difficile* (Supplementary Table S2).

## 4 Discussion

GPI anchors are complex glycolipid structures that covalently tether proteins to the extracellular surface of cellular membranes. The *pig-L* gene encodes an enzyme involved in catalyzing the second step of GPI anchor biosynthesis, playing a role in various biological and pathological processes associated with GPI anchors. This study first analyzed the homology of the CD23150 (*pig-L*) gene with GPI anchors using sequence alignment methods. Subsequently, CRISPR-Cas9 technology was employed to construct ∆*pig-L* and its complementation strain, ::*pig-L*. Gene and expression level assays confirmed successful construction of the knockout and complementation strains. Growth rate analysis revealed no significant differences in lag, exponential, or stationary phases between ∆*pig-L*, :: *pig-L* mutants, and WT strains; however, during the decline phase, mutant strains exhibited significantly reduced autolysis rates compared to WT. The ∆*pig-L* strain showed a marked decrease in toxin gene expression levels and cytotoxicity, with no significant change in biofilm formation ability. This strain also exhibited significantly reduced resistance to reactive oxygen species (ROS). Proteomic analysis revealed that, compared to the WT strain, 170 proteins were significantly upregulated, and 101 proteins were downregulated in ∆*pig-L* mutants. These strains exhibited noticeable metabolic remodeling in fatty acid utilization, endospore resistance, polysaccharide degradation, and anaerobic fermentation. Downregulation of ion homeostasis and nucleotide metabolism suggested altered stress response strategies.

Toxin gene expression assays and cytotoxicity experiments indicated that the ∆*pig-L* mutant strain had significantly reduced toxin expression and cytotoxicity levels. This phenotype is consistent with observations from CD27900 (another PIG-L family member) knockout strains, which showed decreased autolysis rates, virulence, and tolerance to acid and antibiotics (Yang et al., 2023). Moreover, we observed a 1511.49-fold decrease in the expression of Cholera toxin secretion protein E (EpsF), an essential inner membrane platform protein in *Vibrio cholerae*’s T2SS involved in cholera toxin secretion (Abendroth et al., 2009; Shannon et al., 2025). This drastic reduction may correlate with decreased extracellular toxin levels in *C. difficile*.

Biofilm formation experiments showed that the ∆*pig-L* mutant had a slightly lower biofilm formation capacity than WT, without significant differences. Interestingly, the :: *pig-L* complementation strain significantly enhanced its biofilm formation ability compared to WT, suggesting *pig-L* positively regulates *C. difficile* biofilm formation. Proteomic analysis revealed a downregulation of about 2-fold in sugar PTS system EIIA component (crr, K02777) protein. Crr, as part of the Sugar PTS system, typically upregulates *Klebsiella pneumoniae* biofilm formation through the cAMP-CRP signaling pathway by increasing the synthesis of Type 3 fimbriae. The ∆*crr* mutant shows reduced Type 3 fimbriae expression and significantly decreased biofilm formation due to weakened cAMP-CRP signaling.

The ∆*pig-L* strain also demonstrated significantly reduced tolerance to oxidative stress compared to WT. Proteomic analysis showed a decrease of 1.57- and 1.30-fold in *cooS* (carbon monoxide dehydrogenase catalytic subunit) and *gcvH* (GCSH oxidoreductase complex), respectively, both possessing oxidoreductase activity (Qiuying et al., 2022; Lingna et al., 2021). These proteins collectively form a “carbon-electron-antioxidant” defense system against ROS damage (Lu and Imlay, 2021). The decreased expression of these proteins may explain the reduced H_2_O_2_ tolerance in ∆*pig-L* mutants.

Despite successfully constructing ∆*pig-L* and :: *pig-L* strains via CRISPR-Cas9, this study has limitations. Firstly, while it is speculated that *pig-L* affects biofilm formation possibly through *crr* gene regulation, direct experimental validation, such as *crr* knockout or overexpression assays, was lacking. Secondly, while key proteins like *cooS*, *acsA*, and *gcvH* were downregulated in oxidative stress tolerance studies, their precise roles and impacts require further investigation. Lastly, experimental results mainly focused on *in vitro* conditions, lacking *in vivo* validation (e.g., animal models), which limits comprehensive understanding and application.

The findings of this study offer new insights into the biological characteristics and pathogenic mechanisms of *C. difficile*, with potential applications. Firstly, elucidating *pig-L’*s role in toxin production and biofilm formation provides a basis for developing novel therapeutic strategies against *C. difficile* infections, such as designing small-molecule inhibitors targeting *pig-L* or its downstream regulatory pathways to reduce biofilm formation and toxin production. Secondly, research on *pig-L*’s involvement in oxidative stress tolerance presents potential targets to boost bacterial resistance to oxidative stress, enhancing their application value in biotechnology. Finally, this study deepens our understanding of *pig-L*’s gene function, laying a foundation for further control of CDI infections.

## Data Availability

The datasets presented in this study can be found in online repositories. The names of the repository/repositories and accession number(s) can be found below: http://www.proteomexchange.org/, PXD067439.

## Footnotes

^1^ https://www.ncbi.nlm.nih.gov/

^2^ https://blast.ncbi.nlm.nih.gov/Blast.cgi

^3^ https://www.genome.jp/tools-bin/clustalw

^4^ https://services.healthtech.dtu.dk/services/TMHMM-2.0/
