# Supplementary material for: Pig-L mediates virulence, biofilm formation, and oxidative stress tolerance in Clostridioides difficile
Source: Front Microbiol. 2025 Oct 30;16:1691769. doi: 10.3389/fmicb.2025.1691769 (PMC12613277; doi:10.3389/fmicb.2025.1691769)
Supplement: Supplementary file 1 [file Table_1.DOCX]

Supplementary Material

# 1 Supplementary Tables

# Table S1:

|  | Table S1 Plasmids and Primers |  |
| --- | --- | --- |
| Name | Characteristics and sequences | Origins |
| pMTL82151 | pBP1 ori, CmR, *ColE1* ori, *TraJ*, *lacZ* α fragment | (Heap et al., 2009) |
| pJZ23 | iLacP::AsCas9, *Btg*ZI-*Btg*ZI double sites, “Chassis” plasmid for gene-targeting plasmid construction | (Zhang et al., 2020) |
| pHTY4 | Including the targeting site for the *pig-L* gene, derived from the pZJ23 plasmid | This study |
| pHTY5 | Including the targeting site for the *PIG-L* gene and its upstream and downstream homologous arms, derived from the pHTY4 plasmid. | This study |
| pHTY6 | Derived from the CD630 genome, for complementation of the *PIG-L* gene, containing the *mate* gene and its promoter | This study |
| HW2066 | caatcatatcatcaaggagtacac | This study |
| HW2067 | cagtaagcccattcattatg | This study |
| HW2125 | attcgagctcggtacccggggaatccc | This study |
| HW2126 | gcgtgacgtcgactctagagctagggtaatctatcacact | This study |

# Table S2:

Table S2 Top 10 genes with expression changes in WT and ∆*pig-L* strains.

| **ID** | **Gene_ID** | **Name** | **Average expression level** | | **Fold Change** | **Up/Down** |
| --- | --- | --- | --- | --- | --- | --- |
|  |  |  | **WT** | **∆*pig-L*** |  |  |
| 1 | CD05960 | Spore coat associated protein CotJA | 0.1 | 659.32 | 6593.19 | up |
| 2 | CD25460 | Endoglucanase (Cellulase) | 0.1 | 438.64 | 4386.37 | up |
| 3 | CD02940 | ABC-type transport system, bacitracin/multidrug-family permease | 0.1 | 405.96 | 4059.6 | up |
| 4 | CD21020 | Cation:proton antiporter | 0.1 | 251.37 | 2513.71 | up |
| 5 | CD06280 | Putative membrane protein | 2777.16 | 9067.09 | 3.26 | up |
| 6 | CD30970 | PTS system beta-glucoside-specific EIIBCA component | 507.81 | 1438.26 | 2.83 | up |
| 7 | CD14770 | FeoA domain | 123.14 | 345.76 | 2.81 | up |
| 8 | CD14790 | Ferrous iron transport protein B | 120.77 | 331.07 | 2.74 | up |
| 9 | CD03010 | ABC transporter ribose-specific ATP-binding protein | 295.78 | 772.09 | 2.61 | up |
| 10 | CD16260 | D-alanine--D-alanine ligase | 259.64 | 102.29 | 2.54 | up |
| 11 | CD35110 | Cholera toxin secretion protein EpsF | 151.16 | 0.1 | 1511.49 | down |
| 12 | CD17240 | DUF3795 domain-containing protein | 132.76 | 0.1 | 1327.67 | down |
| 13 | CD27230 | Phosphatidylglycerol lysyltransferase | 107.73 | 0.1 | 1077.35 | down |
| 14 | CD06210 | Uncharacterized protein | 106.38 | 0.1 | 1063.72 | down |
| 15 | CD16040 | ABC transporter, ATP-binding protein | 56.12 | 0.1 | 561.17 | down |
| 16 | CD13170 | SAMEA1710456_00375 | 317.77 | 117.01 | 2.72 | down |
| 17 | CD32710 | Spo0E like sporulation regulatory protein | 455.69 | 170.58 | 2.67 | down |
| 18 | CD19240 | Ethanolamine transport protein | 232.28 | 89.04 | 2.61 | down |
| 19 | CD35840 | ABC-type transport system, permease | 773.24 | 378.82 | 2.041 | down |
| 20 | / | Uncharacterized protein OS=*Clostridioides difficile* | 159.07 | 81.06 | 1.96 | down |

# Table S3:

Table S3 Analysis of Minimum Inhibitory Concentrations for different *C.difficile* mutants.

| Antibiotics | Minimum Inhibitory Concentrations of Strains (μg/mL) | | |
| --- | --- | --- | --- |
|  | WT | ∆*pig-L2* | ∷*pig-L2* |
| Amoxicillin | 2 | 2 | 2 |
| Ampicillin | 4 | 4 | 4 |
| Metronidazole | 1 | 1 | 1 |
| Norfloxacin | 16 | 16 | 16 |
| Tetracycline | 32 | 32 | 32 |
| Vancomycin | 2 | 2 | 2 |
| Clindamycin | 32 | 32 | 32 |
| Erythromycin | 128 | 128 | 128 |

**Supplementary References:**

Heap, J. T., Pennington, O. J., Cartman, S. T., and Minton, N. P. (2009). A modular system for *Clostridium* shuttle plasmids. *Journal of Microbiological Methods* 78, 79–85. doi: 10/cs573n

Zhang, J., Hong, W., Guo, L., Wang, Y., and Wang, Y. (2020). Enhancing plasmid transformation efficiency and enabling CRISPR‐Cas9/Cpf1‐based genome editing in *Clostridium tyrobutyricum*. *Biotechnology and Bioengineering* 117, 2911–2917. doi: 10.1002/bit.27435
